# A Single Sex Pheromone Receptor Determines Chemical Response Specificity of Sexual Behavior in the Silkmoth *Bombyx mori*

**DOI:** 10.1371/journal.pgen.1002115

**Published:** 2011-06-30

**Authors:** Takeshi Sakurai, Hidefumi Mitsuno, Stephan Shuichi Haupt, Keiro Uchino, Fumio Yokohari, Takaaki Nishioka, Isao Kobayashi, Hideki Sezutsu, Toshiki Tamura, Ryohei Kanzaki

**Affiliations:** 1 Research Center for Advanced Science and Technology, University of Tokyo, Tokyo, Japan; 2 Transgenic Silkworm Research Unit, National Institute of Agrobiological Sciences, Tsukuba, Japan; 3 Department of Earth System Science, Faculty of Science, Fukuoka University, Fukuoka, Japan; 4 Institute for Advanced Biosciences, Keio University, Tsuruoka, Japan; University of Cambridge, United Kingdom

## Abstract

In insects and other animals, intraspecific communication between individuals of the opposite sex is mediated in part by chemical signals called sex pheromones. In most moth species, male moths rely heavily on species-specific sex pheromones emitted by female moths to identify and orient towards an appropriate mating partner among a large number of sympatric insect species. The silkmoth, *Bombyx mori*, utilizes the simplest possible pheromone system, in which a single pheromone component, (*E*, *Z*)-10,12-hexadecadienol (bombykol), is sufficient to elicit full sexual behavior. We have previously shown that the sex pheromone receptor BmOR1 mediates specific detection of bombykol in the antennae of male silkmoths. However, it is unclear whether the sex pheromone receptor is the minimally sufficient determination factor that triggers initiation of orientation behavior towards a potential mate. Using transgenic silkmoths expressing the sex pheromone receptor PxOR1 of the diamondback moth *Plutella xylostella* in BmOR1-expressing neurons, we show that the selectivity of the sex pheromone receptor determines the chemical response specificity of sexual behavior in the silkmoth. Bombykol receptor neurons expressing PxOR1 responded to its specific ligand, (*Z*)-11-hexadecenal (Z11-16:Ald), in a dose-dependent manner. Male moths expressing PxOR1 exhibited typical pheromone orientation behavior and copulation attempts in response to Z11-16:Ald and to females of *P. xylostella*. Transformation of the bombykol receptor neurons had no effect on their projections in the antennal lobe. These results indicate that activation of bombykol receptor neurons alone is sufficient to trigger full sexual behavior. Thus, a single gene defines behavioral selectivity in sex pheromone communication in the silkmoth. Our findings show that a single molecular determinant can not only function as a modulator of behavior but also as an all-or-nothing initiator of a complex species-specific behavioral sequence.

## Author Summary

Like many animal species, moths use chemical signals called sex pheromones to communicate with conspecific individuals of the opposite sex in the context of reproduction. Typically, male moths depend on sex pheromones emitted by conspecific females to identify and locate their mates. Therefore, the behavioral preference of male moths to conspecific pheromones is a critical factor for successful reproduction. Sex pheromone receptor proteins expressed in specialized antennal olfactory receptor neurons reportedly play a central role in sex pheromone discrimination. However, the causal relationship between sex pheromone receptor specificity and behavioral preference remains to be proven. We have addressed this question in a genetically tractable moth species, the silkmoth (*Bombyx mori*), because this species possesses the simplest possible pheromone system in which a single pheromone substance, bombykol, elicits full sexual behavior. Using transgenic silkmoths expressing a sex pheromone receptor from another moth species, we revealed that solely the chemical specificity of the odorant receptors in bombykol receptor neurons determines the behavioral preference in male silkmoths. Our results show that the initiation of a complex programmed sexual behavior can depend on the properties of a single pheromone receptor gene expressed in a population of olfactory receptor neurons.

## Introduction

In insects and other animals, intraspecific communication between individuals of opposite sex is mediated in part by chemical signals called sex pheromones. In most moth species, male moths heavily rely on species-specific sex pheromones emitted by female moths to identify and orient towards an appropriate mating partner among a large number of sympatric insect species [1]–[3]. The characterization of the genes responsible for behavioral preference in male moths provides a molecular tool for deciphering the genetic mechanisms underlying pheromone-mediated mate recognition.

Sex pheromone signals are detected by male-specific antennal olfactory receptor neurons (ORNs) narrowly tuned to conspecific pheromones and processed by the central nervous system. Using rare males of the European corn borer *Ostrinia nubilalis* or the cabbage looper moth *Trichoplusia ni* that have different pheromone preference from normal males, previous studies reported a correlation between the responsiveness of ORNs and the behavioral preference [4], [5]. Furthermore, using *O.nubilalis* males of two strains that have behavioral preferences for opposite ratios of two pheromone components (*Z*)-11- and (*E*)-11-tetradecenyl acetate, Kárpáti et al. reported that in both strains, ORNs tuned to the major component, regardless its chemical identity, targeted the same morphologically identified region in the brain, concluding that differences in pheromone preference are determined at the level of the ORNs [6].

So far, extensive research has elucidated the molecular mechanisms of pheromone reception that involve several molecular components, such as pheromone binding proteins (PBPs), sensory neuron membrane proteins, Or83b family proteins, and sex pheromone receptor proteins [7], [8]. The selectivity of pheromone receptor neurons is likely to be determined by sex pheromone receptors, because heterologous expression of sex pheromone receptors from several moth species with an Or83b family protein in *Xenopus* oocytes confers specific responsiveness that resembles the specificity of the corresponding pheromone receptor neurons [9]–[12]. In addition, ectopically expressed BmOR1 sex pheromone receptors from *Bombyx mori* or HR13 from *Heliothis virescens* in *Drosophila melanogaster* ORNs also induced responses to their corresponding pheromones, confirming that sex pheromone receptors contain a binding site for pheromones [13], [14]. These observations suggest that sex pheromone receptor genes are strong candidates for determining behavioral preference in male moths. Indeed, using quantitative locus trait analysis, a recent study has reported that male pheromone preference is correlated with a single locus containing at least four sex pheromone receptors in heliothine moths [15]. However, direct evidence that relates the molecular function of sex pheromone receptors in moths to behavioral preference has not been provided so far.

The silkmoth, *Bombyx mori*, is a lepidopteran model insect amenable to genetic manipulation and transgenesis, and is a useful model for characterizing the genes responsible for pheromone preference because this species possesses the simplest possible pheromone system, in which a single pheromone component, (*E*, *Z*)-10,12-hexadecadienol (bombykol), is sufficient to elicit full sexual behavior that includes pheromone orientation behavior and copulation attempts by male silkmoths [16]–[18]. Female silkmoths also emit (*E*, *Z*)-10,12-hexadecadienal (bombykal), which cannot initiate but only negatively modulates components of sexual behavior [19]. Bombykol is detected by the sex pheromone receptor BmOR1, which is tuned specifically to bombykol and is expressed in specialized ORNs in the long sensilla trichodea on the antennae of male silkmoths [9], [20]. Because the tuning of BmOR1 corresponds to a behavioral phenotype, we hypothesized that the ligand specificity of the sex pheromone receptor would determine the behavioral preference, dictating which pheromone chemicals male silkmoths respond to.

In this study, in order to test our hypothesis, we generated transgenic silkmoths expressing the pheromone receptor gene from another moth species in bombykol receptor neurons. Ectopic expression of PxOR1, a sex pheromone receptor from the diamondback moth *Plutella xylostella*, conferred both physiological and behavioral responses to its specific ligand (*Z*)-11-hexadecenal. Further, we revealed that projection patterns of transformed bombykol receptor neurons were identical to those of control animals. These results provide evidence that activation of bombykol receptor neurons alone is sufficient to trigger full sexual behavior. Consequently, the ligand specificity of the pheromone receptor in bombykol receptor neurons is responsible for the initiation of sexual behavior in the silkmoth.

## Results/Discussion

If pheromone preference and initiation of sexual behavior is indeed solely determined by the sex pheromone receptor gene and resulting ORN activation, introducing another receptor gene should confer modified preference. To examine this, we used the PxOR1 sex pheromone receptor from the diamondback moth, *P. xylostella* [10]. Female *P. xylostella* produce a blend of sex pheromones with (*Z*)-11-hexadecenal (Z11-16:Ald) and (*Z*)-11-hexadecenyl acetate (Z11-16:Ac) as major components, and (*Z*)-11-hexadecenol (Z11-16:OH) as a minor component [21], [22]. PxOR1 was identified as a receptor for Z11-16:Ald, based on its ability to specifically confer electrophysiological responsiveness to Z11-16:Ald in *Xenopus* oocytes when coexpressed with PxOR83 [10], the *P. xylostella* orthologue of the Or83b co-receptor [23], [24]. Coexpression of PxOR1 with BmOR2 [9], [20], the *B. mori* Or83b orthologue, induced dose-dependent responses to Z11-16:Ald in oocytes, although the sensitivity was somewhat reduced compared to oocytes coexpressing PxOR1 and PxOR83 (Figure S1). This confirms, however, that PxOR1 forms a functional heteromeric OR complex with BmOR2 and contains the specific binding site for Z11-16:Ald.

**Figure 1:**
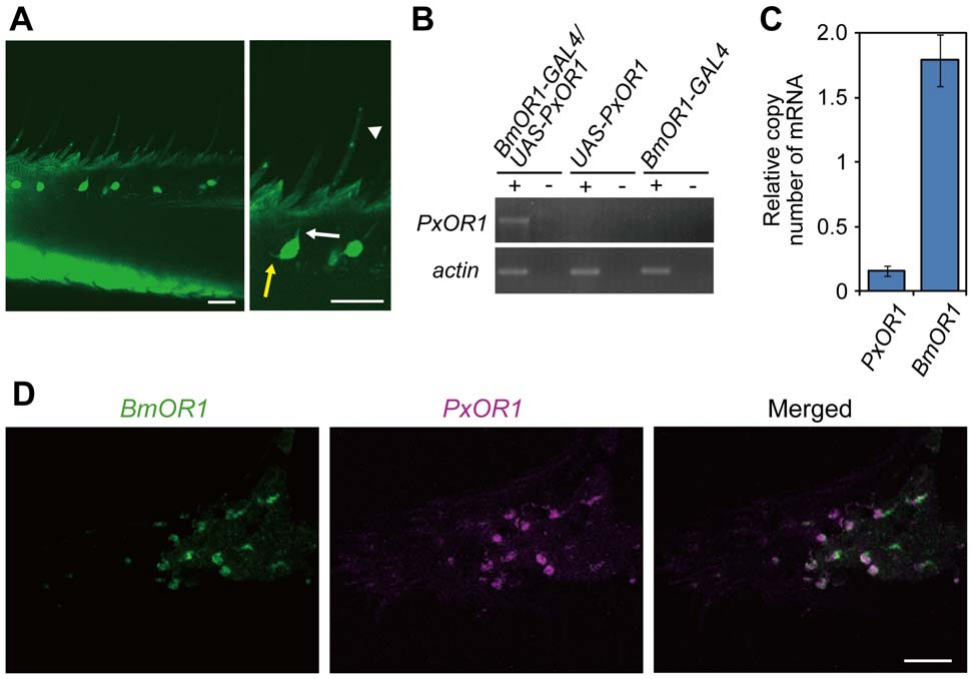
Transgenic silkmoths expressing PxOR1 in bombykol receptor neurons. (A) EGFP expression in the antennae of male moths carrying *BmOR1-GAL4* and *UAS-EGFP* transgenes. Magnified image shows EGFP fluorescence in ORNs innervating pheromone-sensitive long sensilla trichodea. The white and yellow arrows indicate a dendrite and an axon, respectively. The white arrowhead indicates a long sensillum trichodeum. EGFP images were acquired by confocal microscopy (LSM510, Carl Zeiss). Scale bar: 20 µm. (B) PxOR1 expression in the antennae of male moths bearing either *BmOR1-GAL4* and *UAS-PxOR1* transgenes or *BmOR1-GAL4* or *UAS-PxOR1* alone. RT-PCR was performed with RNA isolated from the male antennae of the indicated genotype using *PxOR1*-specific primers. RT-PCR products were separated by electrophoresis. The minus sign indicates that RT-PCR was performed without reverse transcriptase. *B. mori actin1* [52] was used as a positive control in the experiments. (C) The amounts of *PxOR1* and *BmOR1* mRNA in *BmOR1-GAL4*/*UAS-PxOR1* male antennae were determined using quantitative PCR. The data were normalized to the copy numbers of *B. mori rp49* mRNA [54]. Data shown are the means ± SD from three different cDNA pools. (D) Two-color fluorescent *in situ* hybridization of *BmOR1* (green) and *PxOR1* (magenta). Double-labeling was performed on paraffin sections of *BmOR1-GAL4*/*UAS-PxOR1* male antennae using fluorescein-labeled *BmOR1* and DIG-labeled *PxOR1* antisense RNA. Scale bar: 20 µm.

To express PxOR1 in bombykol receptor neurons, we generated a driver line expressing GAL4 under a putative *BmOR1* promoter sequence (*BmOR1-GAL4*) and an effector line expressing PxOR1 under UAS (*UAS-PxOR1*) (Figure S2). Crosses of *BmOR1-GAL4* with *UAS-EGFP* moths [25] revealed that *BmOR1-GAL4* induced enhanced green fluorescent protein (EGFP) expression in ORNs innervating the pheromone-sensitive long sensilla trichodea (Figure 1A). RT-PCR with *PxOR1*-sequence-specific primers revealed that *PxOR1* transcripts were expressed only in the antennae of male moths carrying both *BmOR1-GAL4* and *UAS-PxOR1* transgenes (Figure 1B). Quantitative RT-PCR showed that the copy numbers of *PxOR1* transcripts were about 10 times lower than those of *BmOR1* (Figure 1C). In two-color fluorescent *in situ* hybridization analyses of antennal sections of *PxOR1*-expressing moths, all cells labeled with *PxOR1* cRNA probes were also stained with the *BmOR1* cRNA probes (Figure 1D), indicating that *PxOR1* expression driven by the *BmOR1-GAL4* driver line faithfully recapitulated endogenous *BmOR1* expression.

**Figure 2:**
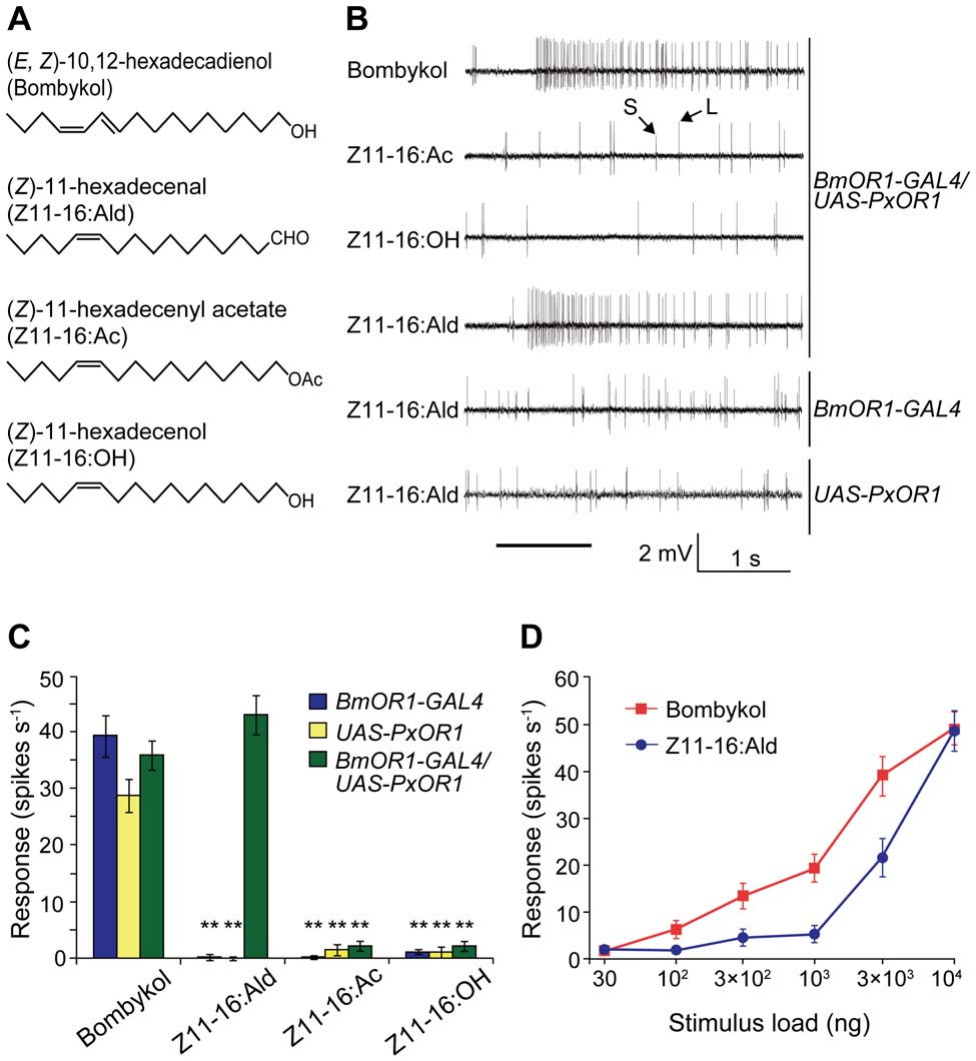
Single sensillum responses of PxOR1-expressing bombykol receptor neurons to Z11-16:Ald. (A) Structure of bombykol and *P. xylostella* pheromone components. (B) Typical electrophysiological recordings from bombykol receptor neurons of transgenic male moths to 10 µg of bombykol or pheromone components of *P. xylostella*. Spikes with large amplitude (L) and small amplitude (S) are from bombykol and bombykal receptor neurons, respectively. The stimulus was applied for 1 s, as indicated by the solid line under the traces of the recordings. (C) The response for 1 s following stimulation with 10 µg of bombykol or pheromone components of *P. xylostella*. Error bars represent ± SEM: *BmOR1-GAL4* (*n* = 14), *UAS-PxOR1* (*n* = 14), *BmOR1-GAL4*/*UAS-PxOR1* (*n* = 12). Two asterisks, *P*<0.01 compared with responses of the corresponding line stimulated with bombykol; Scheffé's F test. (D) Dose-dependent increases in the bombykol (red) or Z11-16:Ald (blue)-induced spike frequency of *BmOR1-GAL4*/*UAS-PxOR1* male moths. Error bars represent ± SEM (*n* = 10).

To examine the effects of ectopically expressed PxOR1 on the electrophysiological properties of bombykol receptor neurons, we carried out single sensillum recording of long sensilla trichodea of male antennae under an airstream containing bombykol, Z11-16:Ald, Z11-16:Ac, or Z11-16:OH (Figure 2A). In addition to a bombykol receptor neuron, each male long sensillum trichodeum comprises one ORN that expresses the receptor for bombykal, named BmOR3 [9], and is sensitive to bombykal [19]. Spikes from these two ORNs are sorted by their amplitudes; the bombykol receptor neuron produces large amplitude spikes, while the bombykal receptor neuron produces small amplitude spikes [19] (Figure 2B). Bombykol receptor neurons expressing PxOR1 responded to Z11-16:Ald and bombykol, but not to Z11-16:Ac or Z11-16:OH (Figure 2B and 2C). Bombykol receptor neurons in males carrying either *BmOR1-GAL4* or *UAS-PxOR1* alone did not respond to any of the *P. xylostella* pheromone components, while robust responses to bombykol were detected in these moths (Figure 2C).

The neural activity induced by Z11-16:Ald was dose-dependent, with a threshold amount of approximately 1 µg on filter paper (Figure 2D and Figure S3). This is about one order of magnitude larger than the threshold amount for bombykol-induced activity (Figure 2D). The lower sensitivity for Z11-16:Ald is probably the result of lower expression of PxOR1 (Figure 1C, see above), although we cannot exclude an effect of the absence of *P. xylostella* PBP [10], which has been reported to enhance sensitivity of ORNs by efficiently solubilizing odorants in aqueous solution [13], [26]. Nonetheless, these results demonstrate that ectopic expression of PxOR1 confers bombykol receptor neurons the ability to respond specifically to Z11-16:Ald. So far, ligand specificities of sex pheromone receptors have been largely examined using heterologous expression systems. When coexpressed with the Or83b family protein in *Xenopus* oocytes, most sex pheromone receptors respond specifically or predominantly to a single pheromone component [9]–[12] of the corresponding species, whereas sex pheromone receptors expressed in modified HEK293 cells require the PBP of the corresponding species for specific responses to pheromones [27]–[29]. This resulted in the hypothesis that PBPs contribute not only to sensitivity but also to specificity of ORNs. Here, we showed that *P. xyllostella* PBP is not necessary to induce a specific response of PxOR1 to Z11-16:Ald in bombykol receptor neurons. The simplest interpretation of this result is that BmorPBP1, a sole PBP known to be expressed in sensilla trichodea of male silkmoths [30], bound and transported Z11-16:Ald to the PxOR1-BmOR2 heteromeric receptor. Indeed, *in vitro* binding analyses of BmorPBP1 to silkmoth pheromones or their analogs have shown that BmorPBP1 possesses the ability to bind a broad range of chemicals [31], [32]. Most importantly, BmorPBP1 has been reported to bind Z11-16:OH [33] which did not elicit responses in bombykol receptor neurons expressing PxOR1, suggesting that the response specificity of pheromone receptor neurons is determined by the response spectrum of the expressed receptor protein in the moth pheromone system.

**Figure 3:**
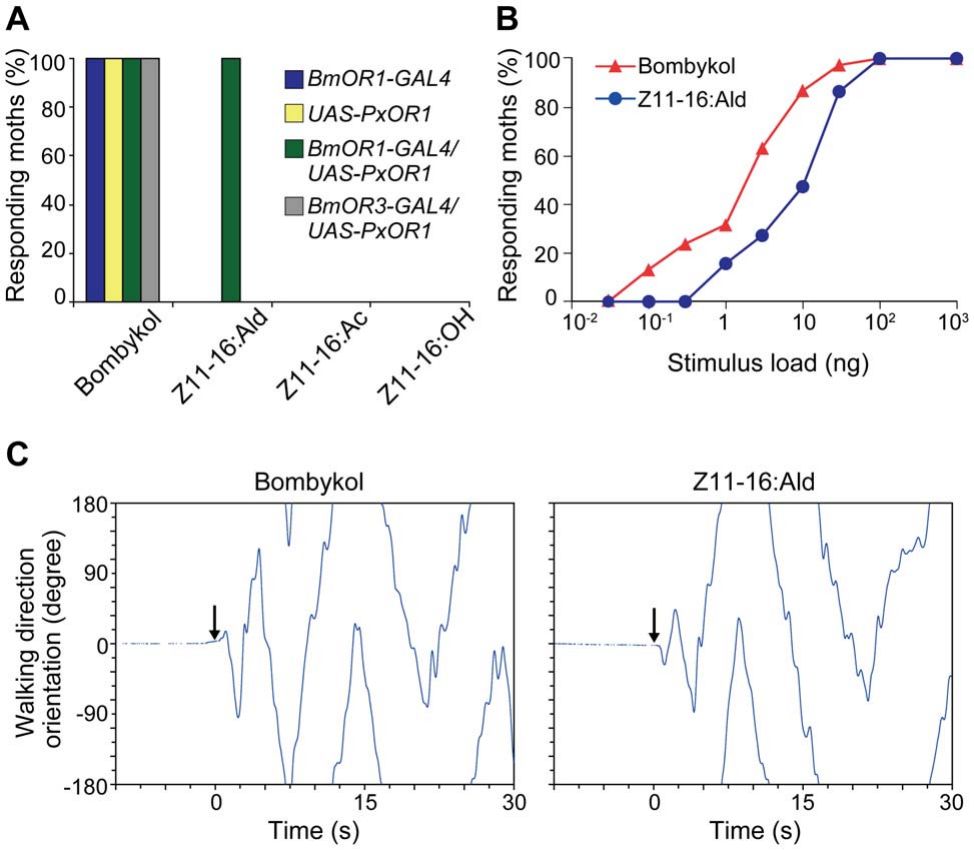
PxOR1-expressing males exhibit pheromone-orientation behavior in response to Z11-16:Ald stimulation. (A) Behaviorally responding percentages of male moths of the indicated genotype. The moths were exposed to 100 ng of bombykol or the pheromones of *P. xylostella*. The display of wing flapping was used as the criterion for a behavioral response to pheromone. The numbers of samples are as follows: *BmOR1-GAL4* (*n* = 13), *UAS-PxOR1* (*n* = 15), *BmOR1-GAL4*/*UAS-PxOR1* (*n* = 38), *BmOR3-GAL4*/*UAS-PxOR1* (*n* = 10). (B) Dose-dependent increase in the percentages of moths that responded to bombykol or Z11-16:Ald (*n* = 22–38). The sensitivity of PxOR1-expressing males to Z11-16:Ald was significantly lower than that to bombykol; GLM, *P*<0.001. (C) Walking direction orientation in a *BmOR1-GAL4*/*UAS-PxOR1* male moth after a single pulsed stimulation (500 ms) of 40 ng of bombykol (right) or Z11-16:Ald (left) to antennae. Stimulus onset (at t = 0) is indicated by arrows, an angle of zero degrees indicates the initial forward direction.

To test whether the artificial activation of bombykol receptor neurons, mediated by PxOR1, elicits sexual behavior, we examined the behavioral responses of PxOR1-expressing moths to Z11-16:Ald. Male *BmOR1-GAL4*/*UAS-PxOR1* moths exhibited wing flapping behavior, which always accompanies pheromone orientation behavior in male silkmoths [17], [34], upon stimulation with Z11-16:Ald or bombykol (Figure 3A, Video S1), but not with the other two pheromone components of *P. xylostella* (Figure 3A). On the other hand, males carrying either *BmOR1-GAL4* or *UAS-PxOR1* alone did not show behavioral responses to any of the *P. xylostella* pheromone components. As a control experiment, we generated a driver line expressing GAL4 under a putative *BmOR3* promoter and expressed PxOR1 in bombykal receptor neurons (Figures S2 and S4). None of the males expressing PxOR1 in the bombykal receptor neurons showed behavioral responses to Z11-16:Ald stimulation (Figure 3A), implying that activation of bombykol receptor neurons was necessary and sufficient to trigger pheromone orientation behavior. The dose-response curves of moths expressing PxOR1 in bombykol receptor neurons showed that the sensitivity of the behavioral responses to Z11-16:Ald was about 10-fold lower than that to bombykol (Figure 3B), in agreement with the different sensitivity of PxOR1-expressing bombykol receptor neurons to these two stimuli.

Tracing the orientation of walking direction angle after single-puff stimulation with Z11-16:Ald demonstrated that the moths performed the programmed zigzag behavior typical of pheromone orientation behavior [34] (Figure 3C). We compared the following behavioral parameters, number of turns, the length of the track walked by moths in 30 s after stimulation (total path length), the direct distance between the start and end points of the track walked (direct distance), and path straightness (direct distance/total path length), and detected no significant difference between stimulation with bombykol and Z11-16:Ald (Table 1), indicating Z11-16:Ald elicited normal pheromone orientation behavior in PxOR1-expressing males. Indeed, when exposed to Z11-16:Ald under unrestrained conditions in a wind tunnel, PxOR1-expressing males oriented toward and localized a Z11-16:Ald source as quickly as they localized a source of the same dose of bombykol (56.9±6.1 vs. 62.9±10.0 s for Z11-16:Ald and bombykol, respectively. Mean ± SEM, *n* = 6, *P* = 0.62; two tailed *t*-test, Figure S5). In addition, we found that the filter paper loaded with Z11-16:Ald can release full sexual behavior; PxOR1-expressing males bent their abdomen and attempted to copulate with it (Video S2). Furthermore, PxOR1-expressing males also localized and attempted to copulate with *P. xylostella* females (Video S3). These results demonstrate that changes in the response selectivity of bombykol receptor neurons drastically modified the pheromone preferences in male silkmoths.

**Table 1:** Comparison of the behavioral parameters of PxOR1-expressing male moths.

Parameter	Bombykol	Z11-16:Ald	N	P-value
Number of turns	8.5±0.65	8.75±1.03	4	0.564
Direct distance (mm)	40.1±10.5	32.5±6.8	4	0.465
Total path length (mm)	418.4±70.2	433.3±64.5	4	0.715
Path straightness	0.095±0.014	0.080±0.017	4	0.465

Data are shown as the mean ± SEM. Statistical analysis was performed using Wilcoxon signed-ranks test.

**Figure 4:**
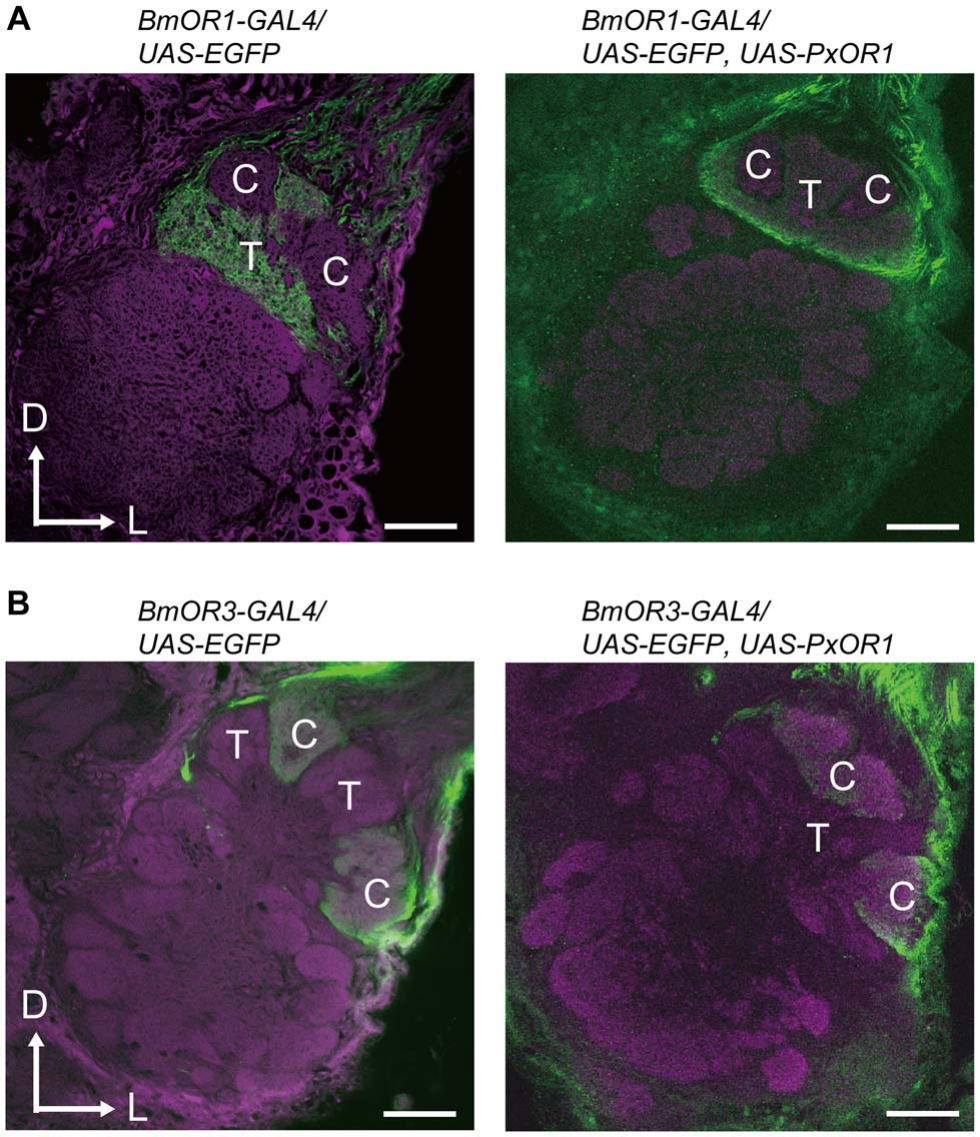
Ectopic expression of PxOR1 does not modify the axonal projections of pheromone receptor neurons. The axon terminals of bombykol (A) or bombykal (B) receptor neurons in the absence (left) or presence (right) of PxOR1 expression were visualized with EGFP followed by anti-GFP immunostaining (green). Background staining was carried out with Alexa Fluor 555 (left) or an anti-synaptotagmin antibody (right) to visualize neuropil structures (magenta). Representative confocal sections are shown. C: cumulus, T: toroid, D: dorsal, L: lateral. Scale bars: 50 µm.

Finally, we asked whether the change in the behavioral response selectivity involves a modification of pheromone processing circuits in the brain. Male moths have a male-specific pheromone-processing structure called the macroglomerular complex (MGC) in the antennal lobe, the first olfactory center in insects [35], [36]. The silkmoth MGC is divided into three subdivisions named toroid, cumulus, and horseshoe [37], [38]. Of these, toroid and cumulus are specialized to exclusively process bombykol and bombykal information, respectively [37]. We first examined the native projection patterns of pheromone receptor neurons using male moths bearing EGFP driven by *BmOR1* or *BmOR3-GAL4* (Figure S4). Axons of BmOR1-expressing neurons terminated in the toroid, while those of BmOR3-expressing neurons projected into the cumulus (Figure 4A and 4B *left*). PxOR1 expression did not change these projection patterns: bombykol and bombykal receptor neurons expressing PxOR1 projected to the toroid and cumulus, respectively (Figure 4A and 4B *right*). These results indicate that changes in receptor protein expression, and consequently changes in the response selectivity of pheromone receptor neurons, do not modify the input pathway of olfactory information to the antennal lobe. This is consistent with findings that insect odorant receptors lack a functional role in axonal targeting of ORNs [39].

Taken together, Z11-16:Ald information mediated by PxOR1 is perceived as indicating the presence of a conspecific female in the brain of the transgenic males, triggering full sexual behavior, indicating that the behavioral preference of males is determined by the specificity of bombykol receptor neurons originating in chemical specificity of sex pheromone receptors. Furthermore, our results demonstrate that the activation of bombykol receptor neurons is sufficient to trigger full sexual behavior in male silkmoths, clearly showing that pheromone information in silkmoths is coded by a labeled line. Similar observations have been reported in the pheromone system of *D. melanogaster*. In this species, activation of a class of ORNs in sensilla trichodea type 1 mediated by OBP76a and Or67d drives a labeled line involving (*Z*)-11-octadecenyl acetate (11-*cis* vaccenyl acetate) as a pheromone that impairs courtship behavior in males and enhances receptivity to courting males in females [14], [40]. However, the overall contribution of pheromones in the courtship behavior of flies is unclear because the display of the behavior relies on multimodal information [41]. In contrast, we show here that a single molecular determinant can not only function as a modulator of behavior but also as an all-or-nothing initiator of a complex species-specific behavioral sequence. Considering the extremely high behavioral sensitivity of male silkmoths to bombykol [18], transgenic silkmoths that express a given odorant receptor in bombykol receptor neurons could be used as highly sensitive biosensors that can detect and localize a wide variety of odorant sources.

In previous attempts to manipulate pheromone receptor neuron input to the antennal lobe, inter-specific transplantation of antennal imaginal discs between two heliothine moth species has been reported [42]–[44]. These studies have shown that the responsiveness of pheromone receptor neurons and the behavioral preference were modified to those of the donors in a fraction of the recipient individuals. However, whole antennae were replaced by donor antennae. In addition, the transplantation also modified the anatomy of the recipient MGC to that of the donor MGC [43], [44]. Therefore, molecular factors responsible for the modification of behavioral preference could not be identified. In contrast, our study introduced a single sex pheromone receptor gene while other molecular components remained unchanged, directly and unequivocally showing that the chemical response specificity of sexual behavior is determined by the sex pheromone receptor in the silkmoth.

Our results indicate that mate recognition of male silkmoths depends on the specificity of the bombykol-BmOR1 interaction. Previously, BmOR1 has been shown to respond to bombykol and also very weakly to bombykal in the *Xenopus* oocyte expression system [9]. The sensitivity to bombykal of oocytes expressing BmOR1 is at least 300 times lower than that to bombykol (threshold concentration of 100 nM for bombykol and 30 µM for bombykal) [9]. Actually, unnaturally high concentrations of bombykal reportedly induce wing flapping behavior in male silkmoths [19]. Apart from the silkmoth pheromones, single sensillum recordings of bombykol receptor neurons have shown that these neurons can be excited by analogs of bombykol with a threshold concentration 100–10,000 higher than for bombykol [45]. High concentrations of these substances may induce wing flapping behavior in the male silkmoth as well. However, considering the much higher concentrations needed to activate bombykol receptor neurons by other chemicals, we think it is reasonable to regard BmOR1 as a highly specific receptor that mediates only bombykol information to elicit sexual behavior at biologically relevant concentrations.

Our results cannot exclude the possibility that other ORs could contribute to the detection and processing of bombykol information. Besides BmOR1 and BmOR3, there are 3 male-specific or male-predominant ORs that possess significant sequence homology with lepidopteran sex pheromone receptors in the genome of the silkmoth [9], [46]. A previous report, however, has shown that these 3 ORs do not respond to bombykol or bombykal at all when expressed in *Xenopus* oocytes [9]. Therefore BmOR1 is most likely the sole receptor that mediates bombykol information in the silkmoth. To conclusively prove this issue, it would be necessary to generate a BmOR1 knock-out silkmoth, which has so far not been possible technically and must be deferred to future research efforts.

Unlike silkmoths, many moth species use blends of pheromones, composed of several components, and the species-specific ratio of blend components is crucial for male orientation to a female emitter [3]. In such a system, more complex processing would be expected in the antennal lobe or higher olfactory processing centers to extract the blend ratio information [47]. To clarify the association of sex pheromone receptors and their corresponding ORNs for initiation of sexual behavior in moths with multi-component pheromone systems, further work will be necessary.

The identification of sex pheromone receptors as the genes responsible for pheromone preference shed light on genetic mechanisms underlying pheromone mediated mate recognition. In addition, the evolution of the sex pheromone communication systems in moths is proposed to play an important role in reproductive isolation and speciation [48]. Comparative analyses of the function of sex pheromone receptors in various moth species will provide clues that will help to unravel the evolution of the molecular mechanism of moth sex pheromone detection, which is likely to be related to moth speciation by creating mating barriers.

## Materials and Methods

### Animals and chemicals

The w1-pnd strain, which is non-diapausing, and has non-pigmented eggs and eyes, was used in this study. Larvae were reared on an artificial diet (Nihon Nosanko) at 25°C on a 16∶8 h (light/dark) light cycle. Synthetic bombykol was provided by Dr. S. Matsuyama of University of Tsukuba, and the pheromone components of *P. xylostella*, including Z11-16:Ald, Z11-16:Ac, and Z11-16:OH, were provided by Shin-Etsu Chemical, Tokyo, Japan.

### Generation of transgenic moths

For the *BmOR1-GAL4* and *BmOR3-GAL4* constructs, approximately 3.7- and 5.8-kb DNA fragments immediately upstream from the initiation codon of each gene were amplified using the polymerase chain reaction (PCR) from the w1-pnd silkmoth genome DNA using LA *Taq* DNA polymerase (Takara) with the following primer pairs: *BmOR1* forward, 5′-AGGCGCGCCAACGCCACCACTCGTCCGGC-3′, *BmOR1* reverse, 5′-CGGGATCCCTTGAAGCTCTGCGAGGATCG-3′, *BmOR3* forward, 5′-AGGCGCGCCCTGCGAGCTAAAGTGCTGAG-3′, *BmOR3* reverse, 5′-TGCTGATCACTACGTAGAGTGTCGGAGCTC-3′. The PCR products were subcloned into the *Asc*I-*Bam*HI site of *pBacMCS-GAL4* [25] to create *pBacBmOR1-GAL4* or *pBacBmOR3-GAL4* (Figure S2). For *UAS-PxOR1*, the entire protein-coding sequence of *PxOR1* was subcloned immediately downstream from the *UAS* of *pBacMCS-UAS* [49] to create *pBacUAS-PxOR1* (Figure S2). Transgenic silkmoths were generated using the *piggyBac*-mediated germ-line transformation method, as described previously [50], [51].

### Reverse-transcription (RT)-PCR

Total RNA was extracted from antennae of male moths 1–5 days after eclosion using TRIzol reagent (Invitrogen), treated with DNase I, and reprecipitated. RNA was reverse transcribed using an oligo(dT) adaptor primer (Takara) and AMV reverse transcriptase (Takara) at 42°C for 35 min. cDNA of *PxOR1* and *B. mori actin 1* [52] was amplified using Ex *Taq* DNA polymerase (Takara) and the primer pairs for *PxOR1* (5′-GCTCTCCCACTTCTTCACCATG-3′ and 5′-TGCTGGAACAGGATCACCGTC-3′) and *B. mori actin 1* (5′-ATGTGCAAGGCCGGTTTCGC-3′ and 5′-CGACACGCAGCTCATTGTAG-3′) with thermal cycling at 94°C for 1 min, then 30 cycles at 94°C for 30 s, 60°C for 30 s, and 72°C for 30 s, followed by 72°C for 10 min. Equal amounts of the PCR products were separated by electrophoresis on 1.5% agarose gels. No PCR products were produced when reverse transcriptase was excluded during reverse transcription, and sequence analysis confirmed the identity of the cDNA products.

### Quantitative real-time PCR

Total RNA was extracted from antennae of male moths 1–3 days after eclosion, and reverse transcribed as described in the RT-PCR section. Real-time quantitative PCR was performed as described previously [53] using a LightCycler 1.5 (Roche) with the appropriate primer pairs for *PxOR1* (5′-GCGTGGAAAAACTCGAAGAC-3′ and 5′-AAGTCCTTCTTCCCCGTGTT-3′), *BmOR1* (5′-CGTATACAGAGGAGGAGTCGAAA-3′ and 5′-AAATCAGAACACTCCAAGAGCAG-3′), and *B. mori ribosomal protein 49* (*rp49*) [54] (5′-CAGGCGGTTCAAGGGTCAATAC-3′ and 5′-TGCTGGGCTCTTTCCACGA-3′). The reaction mixtures for quantitative PCR were prepared using LightCycler FastStart DNA Master SYBR Green (Roche), and PCR was performed according to the manufacturer's instructions. The amounts of each mRNA were calculated, based on cross pointing analysis, with standard curves generated from standard cDNAs. Quantitative measurements were performed in triplicate and the *PxOR1* and *BmOR1* mRNA copy numbers were normalized to that of *rp49* [54] in the same samples.

### *In situ* hybridization

Digoxigenin (DIG)-labeled *PxOR1* and fluorescein-labeled *BmOR1* RNA probes were synthesized from linearized recombinant pGEM-T Easy vectors (Promega) containing the coding sequence of *PxOR1* and *BmOR1*, respectively, using an SP6/T7 transcription kit (Roche) according to the manufacturer's instructions. *In situ* hybridization was performed as described previously [20]. Antennae of 2- to 8-day-old male moths were fixed in 4% paraformaldehyde/PBS overnight at 4°C, dehydrated, embedded in paraffin, and cut into 12-µm sections. After deparaffinizing, the tissue sections were incubated for 16 h at 60°C in 100 µl hybridization buffer containing 500 ng/ml of both DIG-labeled *PxOR1* and fluorescein-labeled *BmOR1* antisense RNA probes. The sections were washed three times for 5 min each in 0.1% Tween 20/PBS (PBST) at 60°C. The hybridization signal was amplified using the TSA Plus Fluorescence System (Perkin Elmer), and according to the manufacturer's instructions. The DIG-labeled probes were visualized using anti-DIG-POD (Roche; 1∶20) with Cy3 tyramides as the substrate, while the fluorescein-labeled probes were visualized using anti-fluorescein-POD (Roche; 1∶20) with fluorescein tyramides as the substrate.

### Immunohistochemistry

Moth brains were stained immunohistochemically as described previously [55]. Briefly, the brains were dissected from the heads and fixed in 4% paraformaldehyde/PBS overnight at 4°C. Then, the brains were washed in PBS containing 0.2% TritonX-100 several times in PBS (PBTX) and pre-incubated with 5% normal donkey serum and 5% normal goat serum in PBTX (PBTX-NDS-NGS) for 3 h at room temperature. Subsequently, they were incubated with rabbit anti-GFP antibody (Molecular probes; 1∶200) and mouse anti-synaptotagmin monoclonal antibody (Developmental Studies Hybridoma Bank; 1∶100) in PBTX-NDS-NGS at 4°C for 3 days. Next, they were washed in PBTX and incubated with Alexa488-conjugated anti-rabbit IgG (Molecular probes; 1∶200) and Cy3-conjugated anti-mouse IgG antibodies (Jackson Immuno Research Laboratories; 1∶200) in PBTX-NDS-NGS at 4°C overnight. Confocal images were captured using a LSM510 confocal microscope (Carl Zeiss).

### Single sensillum recordings

Electrophysiological recordings were performed in a Faraday cage at 25°C. Moths were fixed on an acrylic plate under an Olympus BX50 (500×) microscope. The antennae were held and stabilized by dental wax (GC Corporation, soft plate wax). Action potentials were recorded by inserting an electrolytically sharpened tungsten wire electrode (diameter 0.5 mm, tip approximately 1 µm) into the bases of long sensilla trichodea on the antenna. As a reference electrode, a platinum plate was inserted in the neck of the moth. Odorant stimulation was prepared in *n*-hexane at 1 ng to 1 µg/µl, and 10 µl of the odorant solution were loaded on 1×1 cm^2^ filter papers. The filter papers with odorants were placed inside Pasteur pipettes (Fisher, 13-678-20A). A charcoal-purified and moistened airstream was passed through the glass pipette (0.4 l/min) and directed onto the antenna. The pipettes were placed with the outlet 2 cm from the recording site. The odorants from the pipettes were delivered by puff stimulation and the air speed at the recording site was 1.8–2.0 m/s. The puff stimulation for 1 s was controlled by a solenoid valve (Takasago Electric, Takasago Clean Valve) and electronic stimulator (Nihon Koden, SEN-7203). A suction tube 50 mm in diameter was placed near the animal to remove the odorants after stimulation rapidly and to avoid uncontrolled stimulation by odorants leaking from the glass pipette. The response was band-pass-filtered (50 Hz to 3 kHz) and amplified (Nihon Koden, MEZ-8300). The electrophysiological data were captured with a Digidata1322 interface (Axon Instruments) attached to a PC. The responses were quantified by counting spikes during 1 s following stimulus onset, and subtracting the number of mean spontaneous spikes/s in a 5 s time window prior to stimulation.

### Behavioral experiments

Male silkmoths were used within 2–8 days after eclosion. The moths (up to 6 per experiment) were placed in a translucent cylindrical acrylic closed box (15 cm in diameter and 6.5 cm in height). An air-puff stimulus was used to spread odorants into the box through a 2-mm-diameter hole in the middle of the lid with a Pasteur pipette containing a piece of filter paper with the odorant. A charcoal-purified airstream (1.4 l/min) was passed through a Pasteur pipette and directed into the box. Pulsed odorant stimulation (200 ms duration) was produced by controlling a three-way solenoid valve with an electronic stimulator (Nihon Koden, SEN-7203). The odorants were dissolved in *n*-hexane, and applied to a piece of filter paper (1×2 cm). In the qualitative analysis, the moths were exposed to 100 ng of bombykol, Z11-16:Ald, Z11-16:Ac, or Z11-16:OH, while in the dose-response analyses, the moths were exposed to increasing concentrations of bombykol or Z11-16:Ald (0.03, 0.1, 0.3, 1, 3, 10, 30, 100, and 1000 ng) at 1-min intervals. The air and odorant were removed through an exhaust tube attached to the side of the box 10 s after each puff stimulation. Wing flapping within 10 s of the stimulation and lasting for more than 10 s was counted as a response. The behavioral response of the moths and the pheromone stimulation were recorded with a digital video camera for further analysis.

To analyze details of the locomotor patterns in response to olfactory stimulation, in particular the orientation of the walking direction, the moths were tethered and placed on a Styrofoam sphere floating on an air cushion. The movements of the sphere were recorded using high-speed optical mice connected directly to a computer running a home-made program for data capture and stimulus control. For stimulation, 40 ng of bombykol or Z11-16:Ald in *n*-hexane was applied to a piece of filter paper (0.5×1 cm), which was inserted into a borosilicate glass cartridge (inner diameter 3 mm). Two cartridges were used, placed in front of the left and right antennae. A charcoal-purified humidified airstream was passed through solenoid valves (Takasago Electric, Takasago Clean Valve) controlling the stimulation through the cartridges. The odorants were removed by a continuous flow generated by a suction tube (50 mm diameter) placed behind the moth resulting in a wind speed of 0.5 m/s in front of the moth's head. To prevent stimulant leakage, the air in front of the cartridges was removed by air streams controlled by a second pair of solenoid valves with a speed of >2 m/s perpendicular to the wind direction except when applying stimuli. The moths were exposed to single puffs of bombykol or Z11-16:Ald with 500 ms duration. The angle of the walking direction was calculated from the movements of the sphere by the computer. The initial forward direction defines zero degrees orientation.

### Behavioral experiments in the wind tunnel

A moth was placed in a wind tunnel that had a working section measuring 180 cm long, 90 cm wide, and 30 cm high. Air flow was introduced into the tunnel by negative pressure generated by a voltage-regulated fan. The wind velocity was adjusted to 0.4 m/s. Then, 100 ng of bombykol or Z11-16:Ald were applied to a piece of filter paper (1×2 cm), which was placed in the wind tunnel 1 cm above the floor. To analyze the response to female *P. xylostella*, 12 female *P. xylostella* were placed in a clean acrylic cage and used as the pheromone source. Individual male silkmoths were placed 15 cm downwind from the pheromone source. The response of the male moths was recorded with a digital video camera and used for analysis.

### Statistical analysis

The single sensillum recording responses of different genotypes were compared using one-way analysis of variance followed by Scheffé's F test, using Microsoft Excel 2007 and a commercial macroprogram (Statcel version 2, Seiun-sya). The behavioral sensitivity to different pheromone components was analyzed with the univariate general linear model (GLM), followed by Bonferroni adjustment for multiple comparisons between groups using R software (http://www.r-project.org/). The Wilcoxon signed-ranks test was used to compare the detailed behavioral parameters in response to different pheromone components with R software.

## Supporting Information

Figure S1 Dose-dependent responses of *Xenopus* oocytes coexpressing PxOR1 with PxOR83 or BmOR2 to Z11-16:Ald. Z11-16:Ald was applied sequentially to the same oocyte. Each point represents the averaged current value (± SEM) (*n* = 10). The Z11-16:Ald-induced dose-dependent current increase, with a 50% effective concentration of 0.42 µM and 1.20 µM in oocytes coexpressing PxOR1 with PxOR83 and PxOR1 with BmOR2, respectively. The threshold concentration was approximately 30 nM and 100 nM for oocytes coexpressing PxOR1 with PxOR83 and PxOR1 with BmOR2, respectively. Expression of odorant receptors in oocytes and electrophysiological recordings of the oocytes were carried out as described previously [10].
(TIF)

Figure S2 Schematic diagrams of the *piggyBac* vectors used to generate transgenic silkmoths. *pBacBmOR1-GAL4* (top), *pBacBmOR3-GAL4* (middle), and *pBacUAS-PxOR1* (bottom) were used to generate *BmOR1-GAL4*, *BmOR3-GAL4*, and *UAS-PxOR1*, respectively. *FibL-EGFP* or *DsRed* indicates a screening marker that drives EGFP or DsRed expression in silk glands. IVR, inverted terminal repeats of the *piggyBac* transposon; SV40, SV40 polyadenylation signal; hsp70, *Drosophila* hsp70 polyadenylation signal.
(TIF)

Figure S3 Representative dose-dependent single sensillum responses to bombykol or Z11-16:Ald in *BmOR1-GAL4*/*UAS-PxOR1* male moths. Doses are indicated on the left of each trace. The stimuli were applied for 1 s, as indicated by the solid line below the records.
(TIF)

Figure S4 EGFP expression in the antennae of male moths carrying *BmOR3-GAL4* and *UAS-EGFP* transgenes. Magnified image (right) shows EGFP fluorescence detected in olfactory receptor neurons. The white and yellow arrows indicate a dendrite and an axon, respectively. The white arrowhead indicates a long sensillum trichodeum. Scale bar: 20 µm (left), 10 µm (right).
(TIF)

Figure S5 Comparison of time to localize bombykol or Z11-16:Ald source in *BmOR1-GAL4*/*UAS-PxOR1* males. The male moth was placed 15 cm downwind of a 100 ng bombykol or Z11-16:Ald source in the wind tunnel with wind velocity of 0.4 m/s. Data are shown as mean ± SEM, no significant difference was detected between bombykol and Z11-16:Ald (*n* = 6, *P* = 0.62; two tailed *t*-test).
(TIF)

Video S1 Pheromone orientation behavior of PxOR1-expressing males in response to Z11-16:Ald stimulation. Male silkmoths bearing both *BmOR1-GAL4* and *UAS-PxOR1* transgenes (without marking) displayed wing flapping behavior, a criterion for the display of pheromone orientation behavior in the silkmoth, upon stimulation with 100 ng of Z11-16:Ald (first stimulus). Moths bearing either *BmOR1-GAL4* (green) or *UAS-PxOR1* (red) alone showed no behavior to Z11-16:Ald, although bombykol stimulation (100 ng) (second stimulus) elicited robust wing flapping behavior in all moths. LED lights indicate the timing of pheromone stimulation. In this movie, moths of *GAL4*/*UAS*, *GAL4* driver, and the *UAS* effector line were placed in the same box for presentation purposes. This is an exception; they were normally tested separately for their behavioral responses.
(MOV)

Video S2 Copulation attempts of a PxOR1-expressing male silkmoth to Z11-16:Ald. A piece of filter paper loaded with 1 µg Z11-16:Ald was placed inside a Pasteur pipette and used as the pheromone source. An individual male silkmoth was placed 3 cm downwind from the pheromone source. After the plastic cup that prevents the moth from detecting Z11-16:Ald was removed, the PxOR1-expressing male displayed pheromone orientation behavior and initiated abdominal bending that is the criterion for copulation attempts in male silkmoths.
(MOV)

Video S3 Orientation of a PxOR1-expressing male to females of *P. xylostella*. Male silkmoths bearing both *BmOR1-GAL4* and *UAS-PxOR1* (without marking), *BmOR1-GAL4* (green), and *UAS-PxOR1* (red) were placed in a wind tunnel. Soon after the glass beaker that blocks the spread of pheromones from the female *P. xylostella* was removed, a PxOR1-expressing male initiated pheromone orientation behavior and succeeded in localizing a female *P. xylostella*. Males bearing either *BmOR1-GAL4* or *UAS-PxOR1* alone displayed pheromone orientation behavior only after the glass beaker covering the female silkmoth was removed.
(MOV)
