# Supplementary figures and images for: A Single Sex Pheromone Receptor Determines Chemical Response Specificity of Sexual Behavior in the Silkmoth Bombyx mori
Source: PLoS Genet. 2011 Jun 30;7(6):e1002115. doi: 10.1371/journal.pgen.1002115 (PMC3128102; doi:10.1371/journal.pgen.1002115)

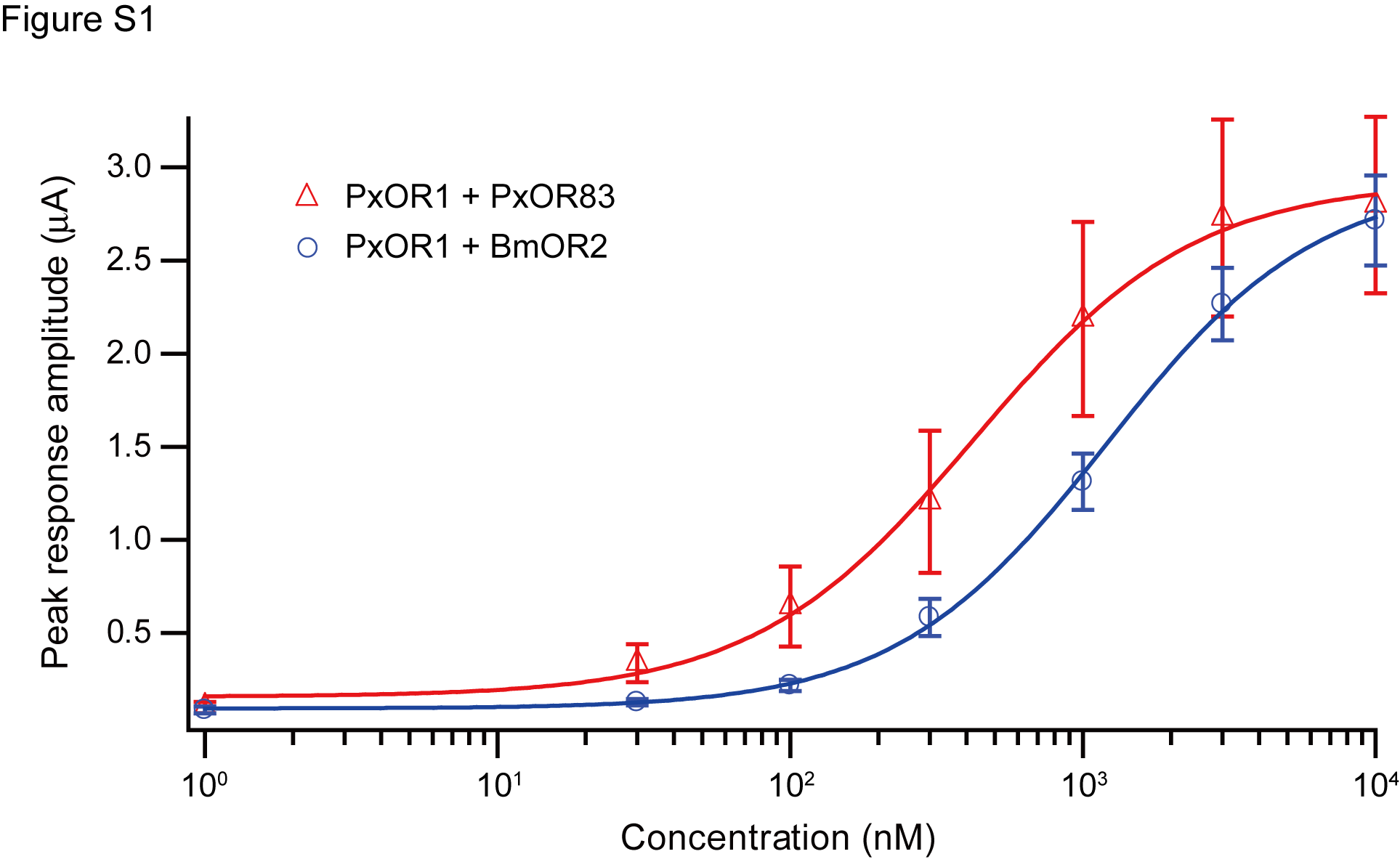

Supplement: Figure S1 — Dose-dependent responses of Xenopus oocytes coexpressing PxOR1 with PxOR83 or BmOR2 to Z11-16:Ald. Z11-16:Ald was applied sequentially to the same oocyte. Each point represents the averaged current value (± SEM) (n = 10). The Z11-16:Ald-induced dose-dependent current increase, with a 50% effective concentration of 0.42 µM and 1.20 µM in oocytes coexpressing PxOR1 with PxOR83 and PxOR1 with BmOR2, respectively. The threshold concentration was approximately 30 nM and 100 nM for oocytes coexpressing PxOR1 with PxOR83 and PxOR1 with BmOR2, respectively. Expression of odorant receptors in oocytes and electrophysiological recordings of the oocytes were carried out as described previously [10]. (TIF) [file pgen.1002115.s001.tif]

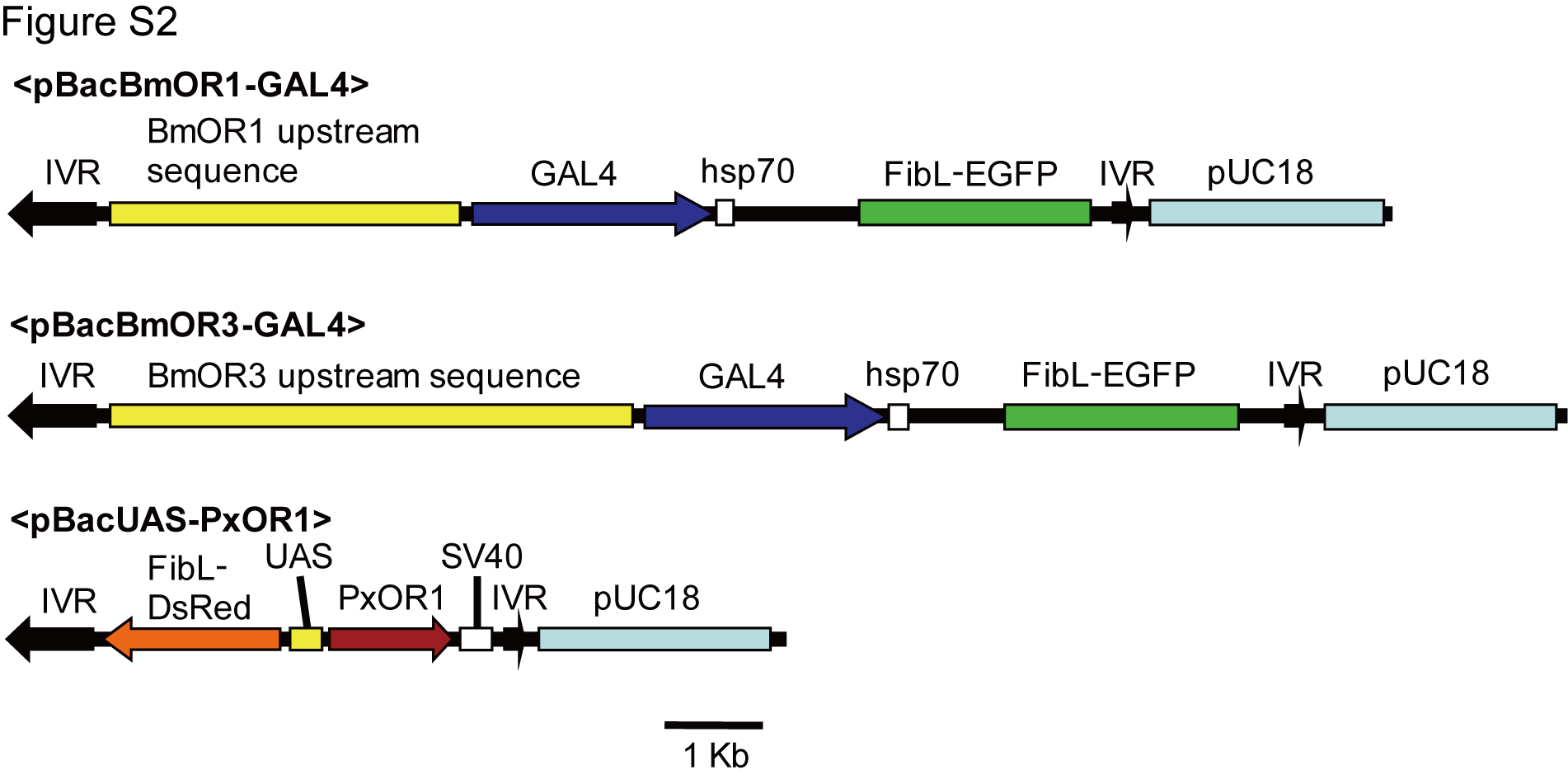

Supplement: Figure S2 — Schematic diagrams of the piggyBac vectors used to generate transgenic silkmoths. pBacBmOR1-GAL4 (top), pBacBmOR3-GAL4 (middle), and pBacUAS-PxOR1 (bottom) were used to generate BmOR1-GAL4, BmOR3-GAL4, and UAS-PxOR1, respectively. FibL-EGFP or DsRed indicates a screening marker that drives EGFP or DsRed expression in silk glands. IVR, inverted terminal repeats of the piggyBac transposon; SV40, SV40 polyadenylation signal; hsp70, Drosophila hsp70 polyadenylation signal. (TIF) [file pgen.1002115.s002.tif]

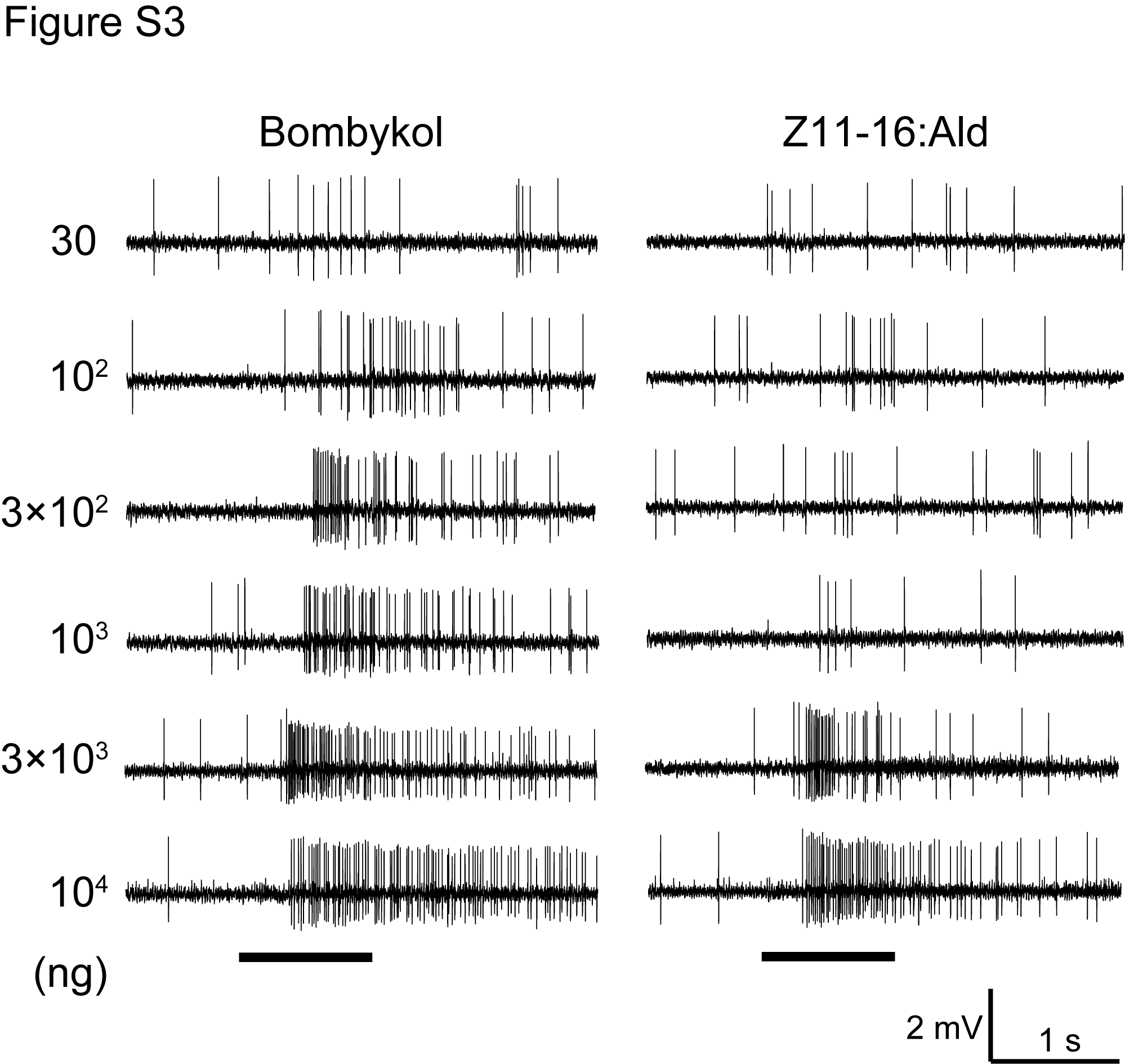

Supplement: Figure S3 — Representative dose-dependent single sensillum responses to bombykol or Z11-16:Ald in BmOR1-GAL4/UAS-PxOR1 male moths. Doses are indicated on the left of each trace. The stimuli were applied for 1 s, as indicated by the solid line below the records. (TIF) [file pgen.1002115.s003.tif]

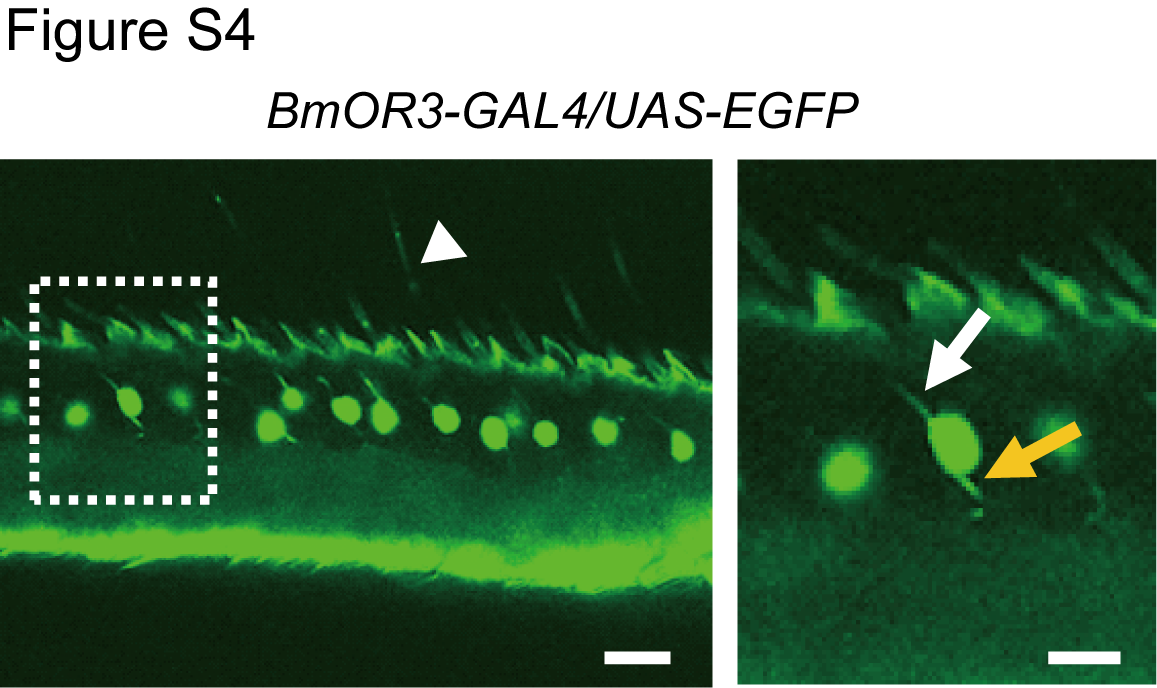

Supplement: Figure S4 — EGFP expression in the antennae of male moths carrying BmOR3-GAL4 and UAS-EGFP transgenes. Magnified image (right) shows EGFP fluorescence detected in olfactory receptor neurons. The white and yellow arrows indicate a dendrite and an axon, respectively. The white arrowhead indicates a long sensillum trichodeum. Scale bar: 20 µm (left), 10 µm (right). (TIF) [file pgen.1002115.s004.tif]

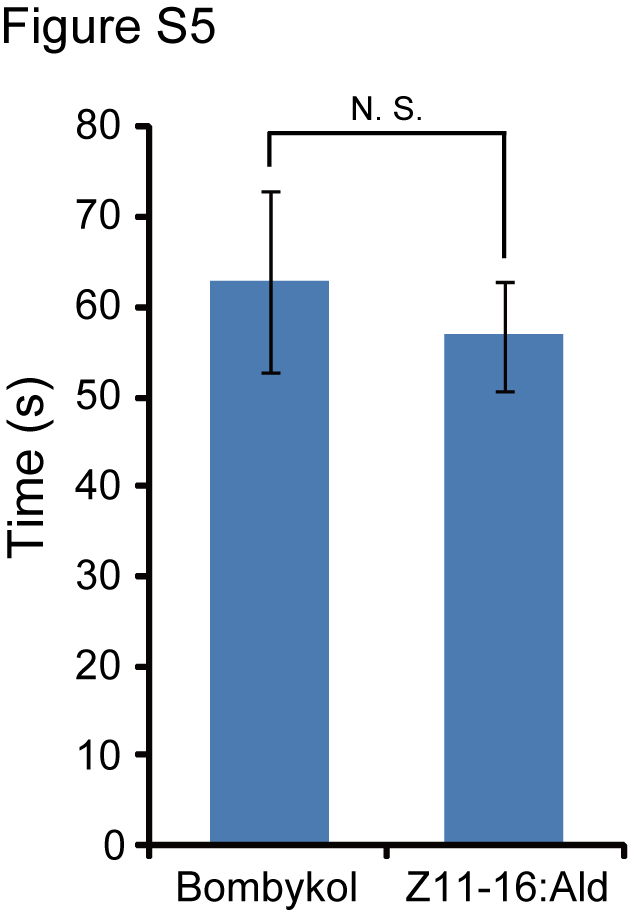

Supplement: Figure S5 — Comparison of time to localize bombykol or Z11-16:Ald source in BmOR1-GAL4/UAS-PxOR1 males. The male moth was placed 15 cm downwind of a 100 ng bombykol or Z11-16:Ald source in the wind tunnel with wind velocity of 0.4 m/s. Data are shown as mean ± SEM, no significant difference was detected between bombykol and Z11-16:Ald (n = 6, P = 0.62; two tailed t-test). (TIF) [file pgen.1002115.s005.tif]
